# Do emotions influence the motivations and preferences of keepers of stingless bees?

**DOI:** 10.1186/s13002-018-0246-3

**Published:** 2018-07-13

**Authors:** Roberta Monique Amâncio Carvalho, Celso Feitosa Martins, Rômulo Romeu Nóbrega Alves, Ângelo Giuseppe Chaves Alves

**Affiliations:** 1 Programa de Pós-graduação em Etnobiologia e Conservação da Natureza, Universidade Federal Rural de Pernambuco, Av. Dom Manoel de Medeiros, s/n – Dois irmãos, Recife, PE 52171-900 Brazil; 2 Centro de Ciências Exatas e da Natureza - Campus I, Departamento de Sistemática e Ecologia, Laboratório de Entomologia, Universidade Federal da Paraíba, Cidade Universitária – Castelo Branco, João Pessoa, PB 58051-900 Brazil; 3 Departamento de Biologia, Universidade Estadual da Paraíba, Av. das Baraúnas, 351 – Bodocongó, Campina Grande, PB 58109-753 Brazil; 4 Departamento de Biologia, Área de Ecologia, Universidade Federal Rural de Pernambuco, Av. Dom Manoel de Medeiros, s/n – Dois irmãos, Recife, PE 52171-900 Brazil

**Keywords:** Ethnoecology, Meliponini, Meliponiculture, Apiculture, Biophilia, Biophobia, Small-scale farming systems

## Abstract

**Background:**

According to the biophilia hypothesis, an emotional affiliation with nature has been inherited during human biocultural evolution. Research on beekeeping can contribute to the scientific understanding of the influence of emotions in the human-nature relationship, since this activity provides concrete experiences of beneficial interaction between the human being and the environment by stimulating conservation-friendly values among practitioners. In this study, we investigated motivations and preferences driving beekeepers’ choices. We hypothesized that emotional criteria would be the main motivators in choosing to include beekeeping into small-scale farming systems. We also assumed that, once beekeeping has been chosen, the preference among species of bees for raising would also be influenced mainly by emotional criteria.

**Methods:**

Data were collected from free lists and semi-structured interviews with 52 keepers of stingless bees from Sítio Xixá in the state of Pernambuco, Brazil. The content analysis technique was used to analyze data from interviews. The underlying criteria for motivation and preference quoted in the free lists were analyzed with Smith’s Salience Index.

**Results:**

Emotional and esthetic criteria were the most salient motivations for choosing beekeeping as one of the activities in small-scale farming systems. On the other hand, honey productivity and bee behavior were the most salient criteria for the preference for certain bee species to be kept.

**Conclusions:**

Emotional criterion had an especially notable influence on the motives for practicing beekeeping, but not on the preference of species to be raised. This demonstrates that the scenario under study represents a panorama of multiple influences in which emotions are one, but not the only, important component. Finally, our results indicate that the emotional domain should be taken into account in environmental education efforts and in the planning of bee management and nature conservation policies.

## Background

Human preferences for particular components of biodiversity, be they species, landscapes, or ecosystems, play an important role in attitudes and behaviors directed toward nature conservation, as well as at the implementation of biodiversity management programs [1–3]. Preferred species may concentrate conservation support over less-preferred species, given that humans usually protect what they consider important to them [4, 5]. Thus, understanding the underlying criteria that influence preferences may reveal useful information for the development of conservation strategies.

Emotions can exert significant influences in the context of human preferences, motivations, and attitudes toward nature [6–8]. The importance of emotions or even the *love* of nature in the predisposition to environmental conservation has been discussed by different authors [9–11]. Wilson [12] denominated biophilia as the innate tendency for humans to associate with the diversity of life and natural processes. With the biophilia hypothesis, Kellert and Wilson [13] suggested that an emotional affiliation with nature has been inherited during human biocultural evolution and, as such, would be linked not only to material exploitation of resources but also to our emotional, esthetic, spiritual, and cognitive development.

Some authors have also studied the influence of emotional orientations on human cognition relative to other animals [14, 15], as well as on the effectiveness of the process of environmental education [16], and emphasized the function of the affective-emotional domain in stimulating human knowledge and learning.

Apparently, the expression of emotional values arising from human-environment interaction depends on direct and continuous contact with nature through beneficial interactions [17, 18]. Therefore, physical and sensorial experience with nature through a beneficial coexistence is fundamental for the cultivation and development of biophilic values.

From this perspective, investigations on activities that provide such experience (e.g., beekeeping) can contribute to the scientific understanding of emotional values that guide preferences, motivations, and human attitudes toward the conservation of nature.

Beekeeping is recognized for contributing to the conservation of pollinator insect populations [19, 20] and for encouraging practices for the maintenance and/or promotion of plant diversity among practitioners, especially around the place of beekeeping, to provide floral resources used in the production of honey and pollen by bees [21, 22]. In this way, beekeeping has the potential to collaborate in reducing the need for deforestation and exploitation of new habitats and natural resources, unlike other intensive and/or conventional farming activities, that imply a greater dependence on the market (e.g., sugar cane monoculture and beef cattle livestock) [23].

Thus, beekeeping may provide concrete experiences of beneficial human-environment interaction by stimulating attitudes of nature conservation among practitioners [24, 25] and potential for sustainable forest management [26].

In Brazil, keeping stingless bees has been a traditional practice among indigenous, afrodescendant communities (generally known in Brazil as “quilombolas”) and other rural populations, especially in the North and Northeast regions [27, 28]. Brazilian bees, called “abelhas sem ferrão” (stingless bees) or “abelhas indígenas” (indigenous bees), compose the Meliponini tribe of Neotropical bees while the activity of raising them is called meliponiculture.

Also noteworthy in Brazil is apiculture, the breeding of poly-hybrids of the genus *Apis* (“honeybees” or “Africanized honeybees”) resulting from the cross between introduced subspecies from Europe (e.g., *Apis mellifera mellifera*) and Africa (e.g., *Apis mellifera scutellata*). Among other characteristics, the use of sting as a defense mechanism differentiates these exogenous bees from Brazilian meliponines, which possess an atrophied sting that is only used as an ovipositor by queen bees [29].

In the present study, we aimed to investigate the criteria underlying motivation and preference among beekeepers. The study was guided by the following questions: (1) What are the motivations considered by local agriculturists in choosing to include beekeeping into their local small-scale farming systems? (2) What species of bees are known and raised by local farmers? and (3) What species of bees are preferred by local farmers and what are the criteria that influence this preference?

Within the context of small-scale farming, where agriculturists commonly use and/or manage a wide range of animal and plant species, we hypothesize that (H1) emotional criteria would be the main motivators in choosing beekeeping as one of the component activities of local small-scale farming systems, and (H2) once this activity has been chosen, the preference among species of bees for raising would also be influenced mainly by emotional criteria.

## Methods

### Study area

**Fig. 1:**
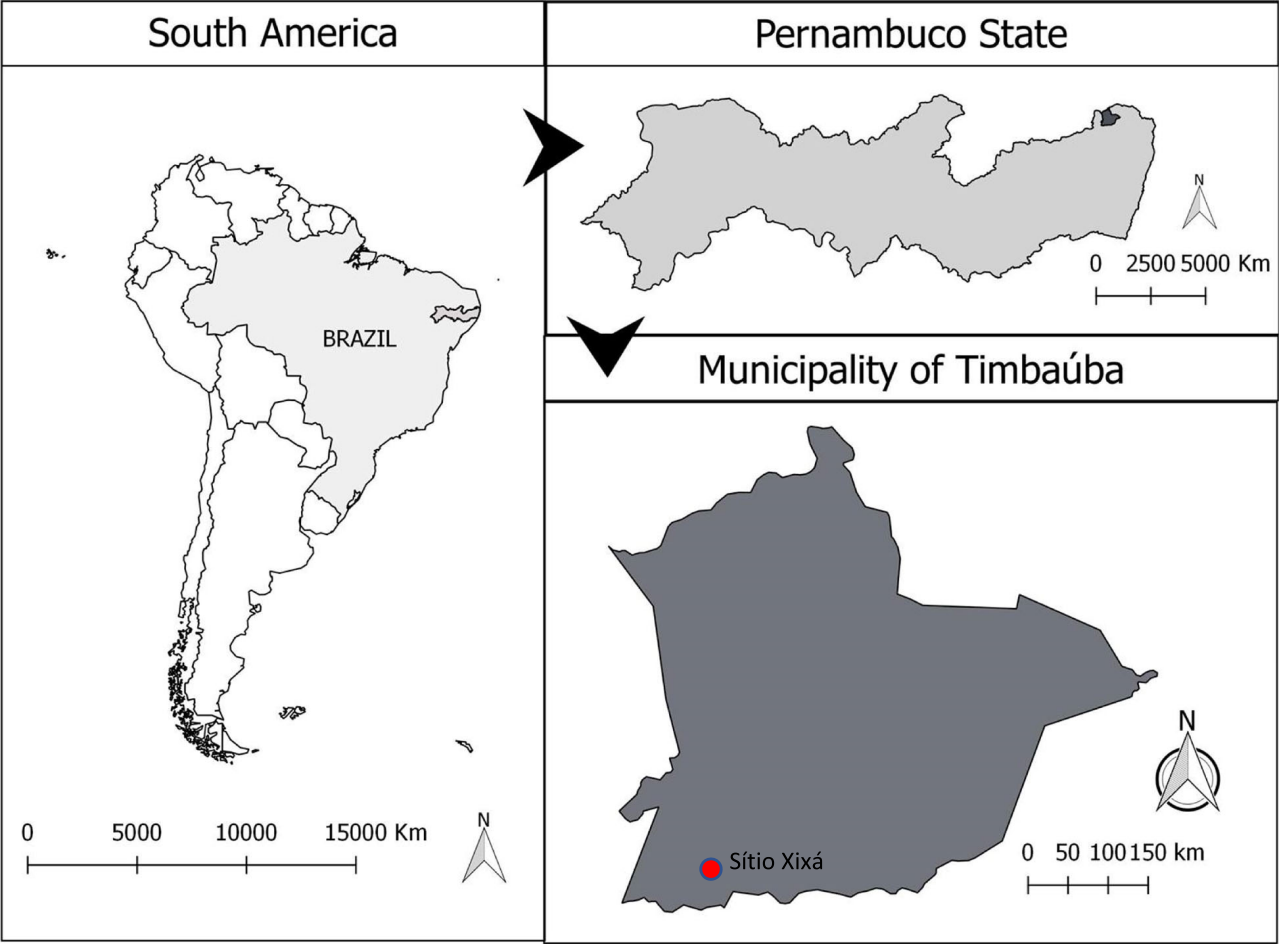
Geographic location of the study area

The research was carried out at Sítio Xixá (07° 35′ 5.96″ S, 35° 24′ 57.66″ W), a rural community in the municipality of Timbaúba, state of Pernambuco, Northeast Brazil. The municipality is located in the Zona da Mata Setentrional Pernambucana (Northern Pernambuco Forest Zone) with an elevation of 101 m (Fig. 1). The estimated population of the municipality is 53,825 inhabitants, of which 14% live in the rural zone, while the remainder lives in the urban zone [30]. The original vegetation is composed of Seasonal Semideciduous Forest and Seasonal Deciduous Forest, ranging up to Dense Montane Ombrophilous Forest. The climate is tropical with a dry season, mean annual temperature ranging from 22 to 26 °C, and mean annual precipitation of 1073 mm [31]. According to Fundação SOS Mata Atlântica [32], the municipality contains approximately 12% of the remaining area of its original Atlantic Forest.

The local economy is based on the sugarcane (*Saccharum officinarum* L.) agroindustry and the production of other crops, such as banana (*Musa* sp.), cassava (*Manihot esculenta* Crantz), beans (*Phaseolus vulgaris* L.), and corn (*Zea mays* L.). Also worth mentioning are livestock raising, footwear and food industries, commercial activities, and handicrafts.

**Fig. 2:**
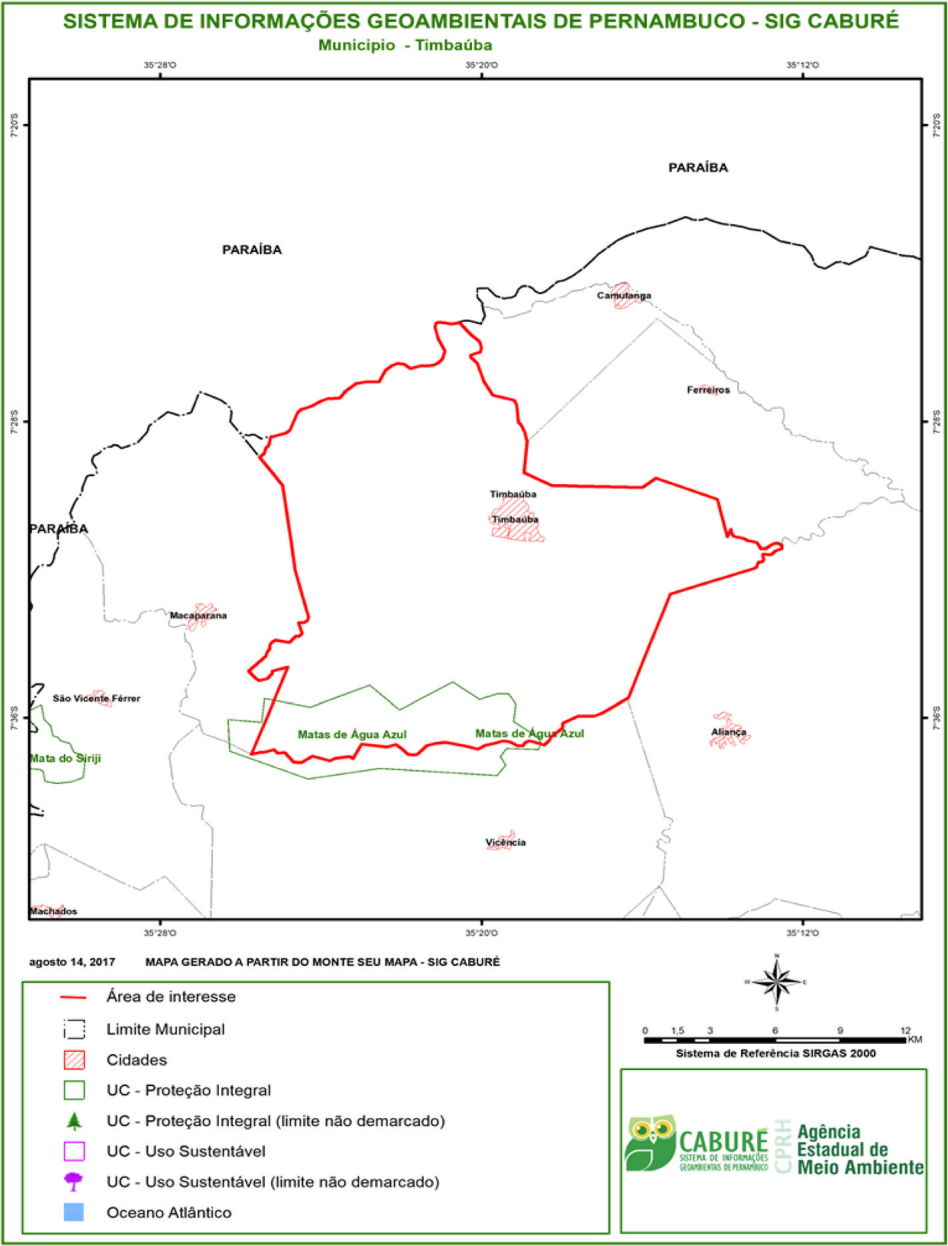
Geographic location of the Matas de Água Azul Wildlife Refuge (natural protected area) in which the study area was carried out. Source: Agência Estadual de Meio Ambiente do Estado de Pernambuco

Sítio Xixá is situated inside a protected natural area called Refúgio de Vida Silvestre “Matas de Água Azul” (“Matas de Água Azul” Wildlife Refuge), which is an Integral Protection Conservation Unit. The conservation unit encompasses a total area of approximately 38 km^2^ and includes portions of three municipalities in the state of Pernambuco: Timbaúba, Vicência, and Macaparana (Fig. 2). Having been created somewhat recently (Decree no. 40.551 of 2014), the conservation unit is still in the process of being implemented, so it lacks a management council and a management plan [31].

According to data from the Municipal Health Secretary, Sítio Xixá has a total of 367 residents, arranged in 105 families. The main source of family income comes from banana (*Musa* sp.) cultivation, but the maintenance of a variety of agricultural small-scale crop fields is a frequent practice for supplementing income or just for self-consumption. Financial aid from government programs also represents an important source of income. Family production is further supplemented by raising animals such as cattle, goats, pigs, and bees. Among the younger generations, it is common to hold temporary jobs in the cities.

### Data collection

The main reason for choosing Sítio Xixá as a study area was the presence of people who were raising bees in small-scale farming systems. Field research was conducted between December 2015 and January 2017. The first contacts with local farmers were intermediated by a technician from the Agriculture Secretary of the municipality. Additional informants were subsequently selected by intentional sampling using the *snowball* technique [33]. Thus, we reached a total of 52 keepers of stingless bees in the study area, which represented approximately half of the local households (45.7%). Among the 52 keepers of stingless bees, only one was also raising Africanized hybrid species of the genus *Apis*.

Among the survey respondents, the majority (88.5%) were men. Age ranged from 27 to 82 years (mean age of 55 years) with 63.5% being older than 50 years. The reported income of the informants was concentrated between one and two times the minimum wage (i.e., between US$ 290 and 580, approximately). As for the level of formal education, 30.8% of the participants were illiterate and, among those having attended school, only two people reported finishing high school.

Prior to data collection, all informants were clarified about the objectives and procedures of the research and only those who confirmed free consent participated. The research project was approved and authorized nationally by the CONEP (National Committee for Research Ethics) through Plataforma Brasil and regionally by CEP-UPE (Research Ethics Committee of the Universidade de Pernambuco) (Protocol CAAE 54357515.7.0000.5207). Authorization to carry out the research was also granted by Agência Estadual de Meio Ambiente (CPRH; State Agency for the Environment), which is responsible officially for the management of protected natural areas in the state of Pernambuco (Process: no. 002434/2017).

Data collection was done using free lists and semi-structured interviews [34]. The interview questions addressed socioeconomic data (age, gender, formal education level, family income, and occupation) and questions related to beekeeping (e.g., How did your interest in beekeeping start? Which kinds of bees do you know? Which kinds of bees do you keep? Which kinds of bees do you prefer to keep?). The free-listing method was applied to specifically obtain motivational (Question: What are your motives for raising bees?) and preferential (Question: Why do you prefer this bee?) criteria. In other words, we analyzed two types of decisions among beekeepers in this research: one regarding “motivations” for choosing beekeeping as part of their family farming system and the other regarding “preferences” in the selection of certain bee species for keeping.

Scientific names of the locally known and kept bee species were determined through the use of entomological collections brought to the field for informant recognition. The collections used belonged to the Laboratory of Entomology of the Universidade Federal da Paraíba, João Pessoa, Paraíba State, Northeast Brazil.

### Data analysis

The interviews were transcribed verbatim and submitted to content analysis [35], from which the categories for analysis regarding the motivation and preference criteria were defined (Table 1). To test the first and second hypotheses, Smith’s Salience Index was used. The Index was calculated with software Anthropac 4.0 [36], determining a salience measure for each criterion, ranging from 0 (minimum) to 1 (maximum). The motivation and preference criteria cited by informants in the free lists were organized into tables using Excel 2013. Each table represented the order of citation of the motivation and preference criteria mentioned by the informants in the free lists. Thus, cultural importance was higher for the criteria that approached the maximum value, i.e., those that obtained higher absolute frequency and ranked first in the free lists obtained from the informants [37].

**Table 1:** Categories for analysis obtained from interviews with informants from Sítio Xixá, state of Pernambuco, Brazil

	Categories for analysis	Terms cited by informants in reference to bees or beekeeping	References
Motivations	Emotional	“like,” “pleasure,” “joy,” “love,” “passion”	Kellert (2012) [18]
	Esthetic	“beautiful,” “ornament,” “beauty”	Kellert (2012) [18]
	Medicinal use of honey	“remedy,” “illness,” “cure”	
	Honey trade	“sell,” “money”	
	Recreation	“diversion,” “relaxation,” “hobby,” “sport”	Kellert (2012) [18]
	Tradition	“family tradition”	
Preferences	Honey productivity	“a lot of honey,” “little honey”	
	Bee behavior	“brave,” “aggressive,” “bites,” “stings,” “meek,” “calm”	
	Honey quality	“dirty honey,” “clean honey,” “dirty bee,” “clean bee”	
	Medicinal potential of honey	“remedy,” “illness,” “cure”	
	Honey price	“sell,” “money”	

## Results

### Motivational criteria for choosing beekeeping

The emotional criterion had the highest values for the salience index (0.638) among the motivations for choosing meliponiculture as one of the activities in family farming systems (Table 2). With regard to economic activity, honey trade had the fourth highest salience index (0.191), followed by esthetics (0.322) and medicinal use of honey (0.274). Recreation (0.109) and family tradition of keeping stingless bees (0.053) followed with lower salience index values. These data reinforce our first hypothesis.

**Table 2:** Salience index of motivational criteria among the informants of Sítio Xixá, state of Pernambuco, Brazil

Motivational criteria	Frequency (%)	Rank	Salience
Emotional	69.2	1.19	0.638
Esthetic	48.1	2.00	0.322
Medicinal use of honey	48.1	2.00	0.274
Honey trade	28.8	2.00	0.191
Recreation	17.3	2.22	0.109
Tradition	5.8	1.33	0.053

### Known and kept bees

The interviewees cited a total of 19 categories of bees known to them, which corresponded to 15 identified scientific species. According to Camargo and Pedro [38], 13 of these species were previously recorded in the state of Pernambuco, and two (*Frieseomelitta dispar* and *Geotrigona* sp.) had only been recorded in other Brazilian states (Table 3).

**Table 3:** Bees known by the informants of Sítio Xixá, state of Pernambuco, Northeast Brazil

Species	Taxonomy (tribe)	Previous occurrence in the state^a^	Local name	Citation frequency (%)
*Apis mellifera*	Apini	Yes	Abelha italiana	100.0
			Abelha africana	88.5
*Frieseomelitta doederleini*	Meliponini	Yes	Moça-branca	63.5
*Frieseomelitta dispar*	Meliponini	No	Mané-de-abreu	57.7
*Geotrigona* sp.	Meliponini	No	Mumbuca ou Munguba	50.0
*Melipona scutellaris*	Meliponini	Yes	*Uruçu boca-de-renda*	100.0
			*Uruçu boca-de-furo*	92.3
*Melipona* sp.	Meliponini	Yes	Mandaçaia	7.7
*Melipona subnitida*	Meliponini	Yes	Jandaíra ou Uruçu-mirim	100.0
*Partamona* sp.	Meliponini	Yes	Cupira	78.8
*Plebeia* sp.	Meliponini	Yes	Abelha-mosquito verdadeira	100.0
			Abelha-mosquito pequena	90.4
*Scaptotrigona* sp.	Meliponini	Yes	Abelha-canudo	53.8
*Scaptotrigona aff. Tubiba*	Meliponini	Yes	Tubiba	75.0
*Tetragonisca angustula*	Meliponini	Yes	Jati	100.0
*Trigona* sp.	Meliponini	Yes	Boca-rasa	67.3
*Trigona* sp.	Meliponini	Yes	Cu-de-vaca	21.1
*Trigona spinipes*	Meliponini	Yes	Aripuá	96.1
Unidentified			Uruçu-preta	15.4
Total	15		19	

^a^Moure’s bee catalog [38]

The species *Apis mellifera*, *Melipona scutellaris*, *Melipona subnitida*, *Plebeia* sp., and *Tetragonisca angustula* were cited by all informants*.*

**Fig. 3:**
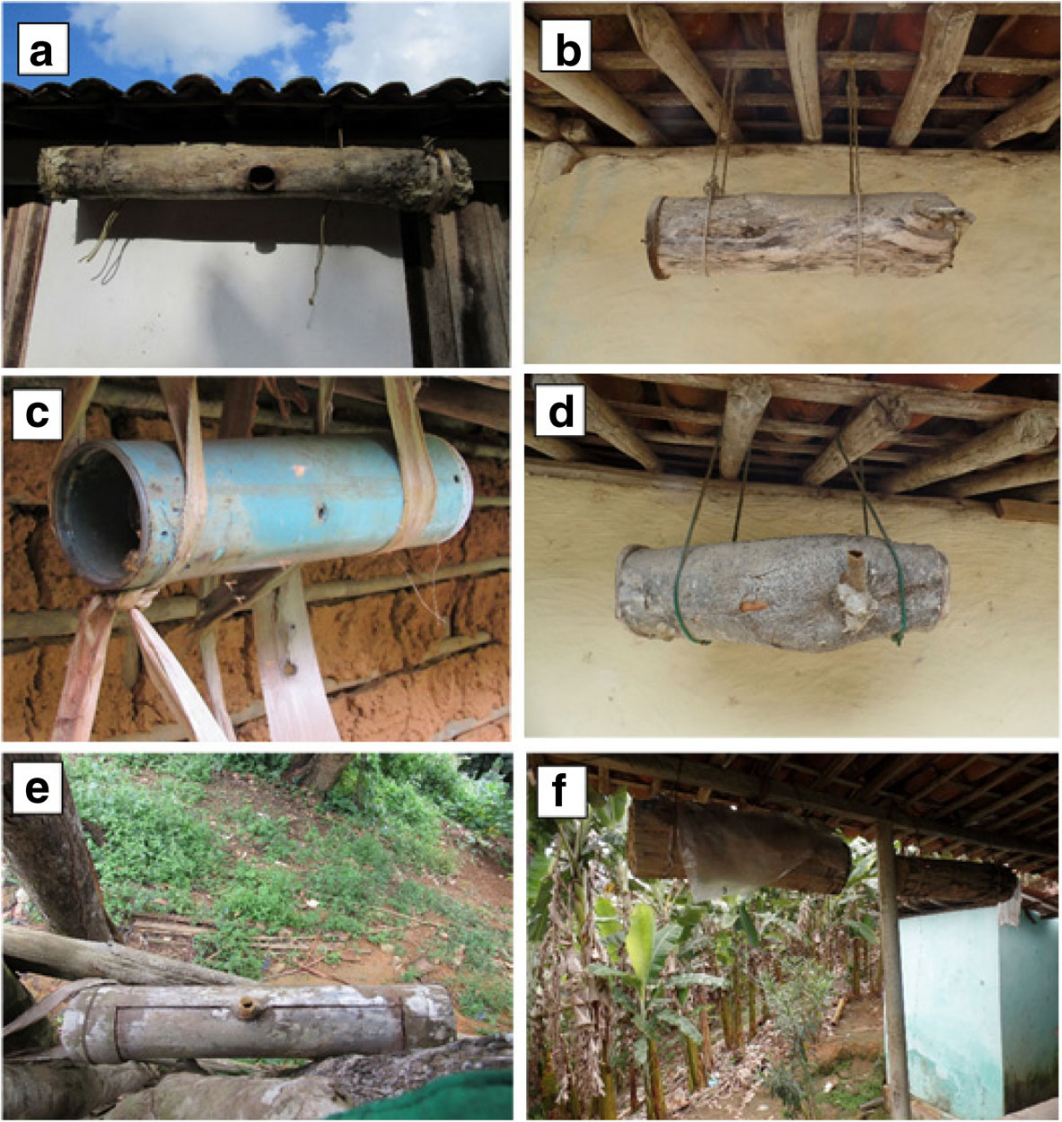
Stingless beekeeping at Sítio Xixá, state of Pernambuco, Northeast Brazil. **a** *Melipona scutellaris*. **b** *Plebeia* sp.. **c** *Tetragonisca angustula*. **d** *Scaptotrigona* sp.. **e** *Scaptotrigona* aff. *tubiba.* **f** *Melipona subnitida*

At least seven species of bees were kept. Of these, six were Neotropical, belonging to the tribe Meliponini: *Melipona scutellaris*, *Plebeia* sp., *Tetragonisca angustula*, *Scaptotrigona* sp., *Scaptotrigona* aff. *tubiba*, and *Melipona subnitida* (Fig. 3). Only one local keeper of stingless bees also kept hybrid Africanized *Apis mellifera*.

All the informants raised *M. scutellaris* and 78.8% concentrated only on this species, while the other 21.2% diversified breeding, ranging from two, three, or even four different species. Even the breeders who had opted for diversification had colonies of *M. scutellaris*, whose honey was used for medicinal and commercial purposes, and few colonies of the other species, generally grown without medicinal or commercial purposes. The exception was that isolated case previously cited in which *A. mellifera* was raised.

### Preference criteria for choice of bee species to keep

All interviewees cited only *M. scutellaris* as the preferred species for beekeeping.

The preference criteria that had the highest salience index (0.716) was honey yield, followed by defensive behavior (0.607) (Table 4). Also the criteria of honey quality (0.3) and the medicinal property of the honey (0.25) were cited. The criterion with the lowest salience index was the price of honey (0.117). These data deny our second hypothesis. Emotional criteria were not directly cited by the informants in expressing their preference for a particular bee species for raising.

**Table 4:** Salience index of preference criteria among the informants of Sítio Xixá, state of Pernambuco, Brazil

Preferential criteria	Frequency (%)	Rank	Salience
Honey productivity	84.6	1.48	0.716
Bee behavior	75.0	1.59	0.607
Honey quality	59.6	2.52	0.300
Medicinal potential of honey	53.8	2.79	0.250
Honey price	25.0	2.69	0.117

## Discussion

### Motivations for keeping bees

#### Emotional and esthetic motivation

Beekeeping probably represents an exemplary case in which the manifestation of biophilic values can be favored to the detriment of economic-financial interests.

Similar to our findings, Yap et al. [39] reported that, according to traditional apiculturists in Northern Vietnam, the observation and the handling of bees provided them with moments “more relaxed” and “happier”. Moore and Kosut [40] noted that watching bees “taking off and returning from their foraging expeditions” was part of the moments of diversion among beekeepers in urban areas. Among the keepers of stingless bees of Sítio Xixá, it was also common to receive reports of moments of diversion and relaxation from observing the foraging habits of bees.

Studying beekeeping in the UK, Maderson and Wynne-Jones [25] discuss “emotional engagement” between beekeepers and bees. According to these authors, regular contact with hives among the most experienced practitioners favored the development of a “multi-sensory sensitivity” toward the natural environment. In that case, the interviewees stated that they had come to feel more intensely certain sounds and scents from the hives and also that the landscapes came to be perceived as habitats and foraging areas for the bees, among other analogous situations. In a similar way, Moore and Kosut [40] reported that beekeepers in some urban areas had an “emotional relationship” with the bees they were keeping.

It seems that beekeeping allows experiences of physical contact and emotional and esthetic appreciation of the natural world, thus providing beneficial interactions that facilitate the expression of biophilic values in the human-environment relationship.

Symbolic aspects also appear to be important in the relationships between humans and bees. Among the keepers of stingless bees in our study, the existence of symbolic representations about *M. scutellaris* was common, especially the attribution of spiritual qualities, wisdom, and capacity of intimate connection between this species and its keepers. Expressions such as “sacred bee,” “divine bee,” and “science bee” were frequent in reference to this species.

Lawrence [41] analyzed symbolic expressions between bees and their keepers and pointed out that because of the display of an extraordinary social structure and the relevant contributions of their products to human benefit, bees have aroused human interest since antiquity. After investigating the association of these insects with a variety of symbolic representations in different cultures, she stated that bees would represent one of the closest interactions mankind has established with nonhuman animals. Yet according to Lawrence [41], the habit of expressing feelings and desires from the symbolic representation of certain animals demonstrates a strong inclination for affiliation on the part of human beings, which would reinforce the symbolic dimension of biophilia.

Thus, as in our results, other scientific reports have indicated that emotional and symbolic values permeate the relationship between beekeepers and bees [25, 39, 40, 42–46]. However, most of these studies put little or no emphasis on emotional values, focusing more on cognitive, material, economic, and even symbolic issues.

Despite the examples previously discussed, it is necessary to emphasize that interactions with bees do not always provide biophilic expressions. For the genus *Apis*, for example, there are reports of both biophilic [25] and biophobic [47] expressions. According to Ulrich [3], biophobia is a partly genetic predisposition to retain feelings of fear or strong negative/avoidance responses to certain natural stimuli, which have been threats during human evolution.

In the case of the study area, the species *M. scutellaris* facilitated the expression of biophilia and the rearing of these insects was mainly related to emotional and esthetic motivations. However, the species *A. mellifera* facilitated expressions of biophobia, leading informants to avoid raising them. In the study area, the biophobic manifestations on *A. mellifera* were explicitly directed to stinging, as well as its production of honey with supposedly few hygienic and medicinal qualities as compared to meliponine honey.

In the study by Cho and Lee [47], school students in South Korea expressed biophobic attitudes toward the genus *Apis* exclusively due to the fear of the sting. However, depending on the socio-ecological context in which it is inserted, this genus can also facilitate biophilic expressions, as reported by Maderson and Wynne-Jones [25] for beekeepers in the UK.

In the case of the American continent, since the introduction of the African bee (*A. mellifera scutellata*) in the state of São Paulo (Brazil) in 1956 [48], Africanized hybrids have spread to the North and South of the continent, having reached the USA in 1990 [49]. Since then, human attacks by these bees in the Americas have been reported [50, 51]. Thus, due to the defensive use of their sting, these bees are often considered aggressive by keepers of native bees and by the general population on the continent and are often referred to as “killer bees” [52].

Thus, it is clear that the genus *Apis* can cause biophobic attitudes to emerge due to its defensive behavior of stinging. On the other hand, we did not find reports of biophobic manifestations in the available literature for the Neotropical bees of the tribe Meliponini.

#### Utilitarian motivation

The criteria for medicinal use and trade honey can be interpreted as utilitarian motivations in beekeeping. Although not as important as emotional and esthetic motivations, these criteria deserve some consideration.

Similar to the results of other authors [53–55], the medicinal home use of honey in the treatment of various diseases in the study area was a more widespread practice than the selling of honey, which happened on a small scale and only occasionally. Thus, most local honey production was regularly reserved for self-consumption, exchanges, and gifts for family and friends.

The medicinal use of honey, usually for personal or family use, reflects aspects of the domestic economy, since the use of this product as an alternative or complement in the local treatment of diseases can probably reduce the expense of conventional medical treatments, thus assuming a role in the household economy.

Among relatives and friends, the regular practice of donating and/or exchanging honey is most often guided by the medicinal value of the product. Similarly, Yap et al. [39] reported that, among Vietnamese beekeepers, 5 to 30% of the honey produced was donated to relatives and friends for the purpose of “strengthen relationships” and “increased respect from the community and relatives.” In this sense, in some societies, the sharing of honey, through exchanges or gifts, seems to be part of a system of local reciprocity in which the maintenance of social bonds does not follow a purely financial perspective. In his classic work, Mauss [56] investigated the relationships of exchanging products in so-called traditional societies and pointed out that the system of giving, receiving, and giving back has constituted one of the fundamental principles of local economic organization and rationale, which sometimes differs from the principles of mercantile exchange conventionally practiced in the West.

In our study, then, the economic issues related to the honey of *M. scutellaris* were more linked to a system of exchange and local reciprocity than to market selling and financial interests.

All of the informants, for example, stated that they had no fixed income from the sale of honey. Thus, even though it was an activity undertaken by nearly half the families, meliponiculture was not among the main local sources of income and the motivations for its accomplishment were more related to emotional and esthetic criteria.

Such cases have also been observed in the relationships of local societies with other biotic components, such as useful plants. After having investigated the main motivations for management practices among the Ixcatec in central Mexico, Rangel-Landa et al. [57] pointed that the maintenance of reciprocal relationships, through donation or exchange, was one of the most important sociocultural factors influencing the management of medicinal and ceremonial plants. In addition, the authors examined the influence of symbolic and esthetic factors on plant management and suggested that these factors were relevant to understanding species management by local human populations.

### Preferred bee species for beekeeping

With regard to bee species preference, the criteria that presented greater salience were related to economic (honey productivity) and ethological (bee behavior) aspects. Preference for bee species for being kept was thus determined by the combination of these two criteria.

Although *A. mellifera* normally has higher honey production than the other locally known bees,^1^ it was not preferred, since it was considered by keepers of stingless bees as an aggressive bee due to its stinging behavior. The species *M. scutellaris*, on the other hand, represented the best combination of preference criteria from the perspective of the informants, since in addition to high honey productivity (among meliponines) it has a less-aggressive behavior (as compared to *Apis* bees), along with better hygienic and medicinal qualities of the honey.^2^

In this scenario, two aspects should be highlighted. The first concerns the criterion of honey productivity. Although this criterion was important, it did not necessarily reflect financial aspects, since the criterion of honey price was the least salient. This result seems to reveal an apparent contradiction. In fact, honey productivity offered from each bee species was a material aspect taken into account by the informants, but not as a direct generator of financial resources, since selling was not the main destination of locally produced honey. As discussed previously, the use of honey in the study area reflected aspects related to household and local economy, rather than macroeconomic and financial aspects.

The second aspect to be emphasized is related to the behavior criterion. Although emotional criterion did not rank high in the preference of informants for bee species, it is important to point out that the behavioral preference criterion was indirectly linked to emotional issues. As pointed out previously, negative emotions, such as fear and aversion, were common toward *A. mellifera* due to its use of sting as a defense behavior. Such negative emotions, therefore, was one of the factors leading local beekeepers to avoid honeybees.

Thus, preference for bee species for being kept in the study area was influenced by the combination of economic (but not necessarily financial) and ethological criteria (which indirectly revealed emotional aspects on the part of the informants).

In other studies, the preference for bee species among beekeepers was also influenced by economic and ethological criteria. Tilahun et al. [58] analyzed the criteria chosen by apiculturists in the selection of colonies of honeybees in northern Ethiopia, and found that bee’s aggressive behavior was considered, but not among the main selection criteria. In that case, even black species with more aggressive stinging behavior were preferred because of their high levels of honey productivity. Contrary to our results, beekeeping among those Ethiopian informants was directed at financially defined interests.

On the other hand, among keepers of stingless bees in Nocupétaro, Mexico [55], the species with the highest levels of importance were those whose products, such as honey and wax, were preferred by local specialists, especially for food, medicine, and in the case of wax, candle production. Thus, similar to our results, the products derived directly from stingless bees were sporadically commercialized by those Mexican beekeepers.

Our results, and the examples cited above [55, 58], illustrate an apparent trend in beekeeping in which meliponiculture is contextually associated with certain cultural values and characterized by a lesser degree of market dependence, while apiculture tended to be practiced from more financially defined purposes [23, 28, 59]. According to this perspective, the expression of economic-financial criteria in the preference of bee species for beekeeping would be more common among apiculturists than keepers of stingless bees. We wonder if the cultural trend of attributing noticeable value to emotional criteria would be maintained by local keepers of stingless bees in a different context, in which the products of stingless bees were explored in a more market-oriented approach. Further studies could provide answers to this additional question.

## Conclusions

Our results suggest that emotions play an important role in human-bee interactions, especially in relation to the motivations for choosing beekeeping as one of the component activities of local farming systems.

Regarding the preference for particular bee species for beekeeping, emotional criterion did not rank high in the responses of the local keepers of stingless bees, although the mentioned criterion of bee behavior seemed to indirectly reveal negative emotions on the part of local beekeepers toward the species *A. mellifera*.

In this way, we noticed a notable influence of emotional criterion on the motivations for beekeeping, but not on the preference of bee species to be raised. Thus, the studied scenario represents a panorama of multiple influences, in which emotions are one of the components, but not the only one. Utilitarian and economic issues also influenced the decisions of local keepers of stingless bees.

Thus, we suggest that future research on the human-bee relationship should include the scientific understanding of emotional values that, in all likelihood, influence (directly or indirectly) the relationship between local populations and the natural environment, together with cognitive, practical, and symbolic components.

In view of the importance of human motivations and preferences in the development of biodiversity management strategies, our results indicate that the emotional domain involving the human-nature relationship must also be taken into account in environmental education efforts [11, 16, 47] and in the planning of bee management and nature conservation policies.

Utilitarian and economic criteria were especially important in relation to the preference for bee species for beekeeping. Nevertheless, in the meliponiculture practiced in the study area these criteria were more related to aspects of the domestic and local economy than to commercial and financial aspects. Beekeeping, especially meliponiculture, seems to represent an exemplary case in which the manifestation of biophilic values can be favored to the detriment of financial and commercial interests. For further inferences, we suggest that future studies approach biophilic and biophobic expressions in human populations that are related to species of native and exotic bees in different socio-ecological contexts.

## Footnotes

1 According to the informants, in the study area, *M. scutellaris* has an average honey productivity of 2 L/colony/year and *A. mellifera* 20 kg/colony/year. Data from the Brazilian Association of Honey Exporters [60] show *A. mellifera* has an average of 15 kg/colony/year. Villas-Bôas [61] recorded 2.2 L/colony/year for *M. scutellaris* in the state of Paraíba, Brazil.

2 According to the informants, *M. scutellaris* would visit only specific flowers in search of resources for honey production, while *A. mellifera* would supposedly visit various types of materials, such as sugarcane resin (see [62]), dead animals, and even animal feces. Thus, honey from *A. mellifera* was considered locally as “dirty” (see [63–65]).
